# Targeted mutation of *BnaMS1*/*BnaMS2* combined with the RUBY reporter enables an efficient two-line system for hybrid seed production in *Brassica napus*

**DOI:** 10.1093/hr/uhae270

**Published:** 2024-09-25

**Authors:** Xiaoxiao Shen, Qing Dong, Xiang Zhao, Limin Hu, Sukanta Bala, Songyue Deng, Yanyan Zhao, Qun Duan, Zilong Liu, Hanzi He, Chuchuan Fan

**Affiliations:** National Key Laboratory of Crop Genetic Improvement, Huazhong Agricultural University, Wuhan 430070, Hubei, China; Hubei Hongshan Laboratory, Wuhan 430070, Hubei, China; National Key Laboratory of Crop Genetic Improvement, Huazhong Agricultural University, Wuhan 430070, Hubei, China; Hubei Hongshan Laboratory, Wuhan 430070, Hubei, China; National Key Laboratory of Crop Genetic Improvement, Huazhong Agricultural University, Wuhan 430070, Hubei, China; Hubei Hongshan Laboratory, Wuhan 430070, Hubei, China; National Key Laboratory of Crop Genetic Improvement, Huazhong Agricultural University, Wuhan 430070, Hubei, China; Hubei Hongshan Laboratory, Wuhan 430070, Hubei, China; National Key Laboratory of Crop Genetic Improvement, Huazhong Agricultural University, Wuhan 430070, Hubei, China; Hubei Hongshan Laboratory, Wuhan 430070, Hubei, China; National Key Laboratory of Crop Genetic Improvement, Huazhong Agricultural University, Wuhan 430070, Hubei, China; Hubei Hongshan Laboratory, Wuhan 430070, Hubei, China; National Key Laboratory of Crop Genetic Improvement, Huazhong Agricultural University, Wuhan 430070, Hubei, China; Hubei Hongshan Laboratory, Wuhan 430070, Hubei, China; National Key Laboratory of Crop Genetic Improvement, Huazhong Agricultural University, Wuhan 430070, Hubei, China; Hubei Hongshan Laboratory, Wuhan 430070, Hubei, China; National Key Laboratory of Crop Genetic Improvement, Huazhong Agricultural University, Wuhan 430070, Hubei, China; Hubei Hongshan Laboratory, Wuhan 430070, Hubei, China; National Key Laboratory of Crop Genetic Improvement, Huazhong Agricultural University, Wuhan 430070, Hubei, China; Hubei Hongshan Laboratory, Wuhan 430070, Hubei, China; National Key Laboratory of Crop Genetic Improvement, Huazhong Agricultural University, Wuhan 430070, Hubei, China; Hubei Hongshan Laboratory, Wuhan 430070, Hubei, China

## Abstract

The recessive genic male sterility (RGMS) method has several benefits in hybrid seed production; however, it is seldom employed in industrial hybrid seed production owing to the difficulty of producing an ample number of pure male-sterile seeds. In this study, we present an efficient methodology for developing a two-line strategy to produce hybrid seed through targeted mutation of *BnaMS1* and *BnaMS2* in conjunction with the *RUBY* reporter in *Brassica napus*. In this method, male-sterile lines were successfully created directly from different elite rapeseed breeding lines through CRISPR/Cas9-mediated mutagenesis and enhanced *Agrobacterium*-mediated transformation. To establish an efficient transgenic maintainer, three seed production technology (SPT) cassettes carrying a functional *BnaMS1* gene linked to different reporters (*DsRed*, *BnaA07.PAP2,* and *RUBY*) were tested and compared in rapeseed. The results indicated that the PMR-based reporter possesses advantages such as phenotypic stability and ease of identification at early stages, making it an ideal tool for rapid and efficient screening. Subsequently, ideal transgenic maintainer lines with a single hemizygous copy of the SPT cassette were successfully developed in the context of *Bnams1Bnams2* double mutants. The progeny from crossing the maintainer line with its male-sterile counterpart exhibited a 1:1 segregation pattern of nontransgenic male-sterile and male-fertile maintainer plants, distinguishable by seedling color. This biotechnological approach to male sterility offers promising prospects for improving the propagation of recessive genic male-sterile plants and the development of hybrid seeds in rapeseed. Furthermore, it is simple to adapt this technique to more *Brassica* crops.

## Introduction

Heterosis has been extensively used to enhance yield, quality, and environmental adaptability in numerous crops [1–3]. Rapeseed (*Brassica napus*), the third largest oilseed crop globally, represents one of the most successful applications of heterosis [4]. The widespread adoption of heterosis has significantly contributed to the swift and extensive surge in rapeseed seed production. Currently, the predominant types of male sterility in competitive hybrid seed production of rapeseed are cytoplasmic male sterility (CMS) and genic male sterility (GMS) [5–7]. The CMS system is cost-effective in producing a fully male-sterile population and is commonly used in rapeseed hybrid seed production [4]. However, developing CMS lines and their maintainer counterparts is typically time-consuming and labor-intensive because of the strict restoring–maintaining relationships required by most CMS systems [8]. In contrast, GMS offers various advantages in heterosis, including stable and complete male sterility, a wide range of restoration characteristics, a shortened breeding cycle, diverse cytoplasmic sources, and the absence of negative cytoplasmic effects [9]. Consequently, GMS shows significant potential for heterosis and has been widely adopted in various systems. Nonetheless, its implementation in hybrid seed production is limited by the difficulty of generating a fully male-sterile population, necessitating the removal of approximately half of the male-fertile plants from female lines once their fertility is detectable, leading to increased hybrid seed production costs. Consequently, GMS shows significant potential for heterosis and has been widely adopted in various systems [10, 11]. The development of environmentally sensitive GMS, such as thermo-sensitive or photoperiod GMS, has expanded the utilization of heterosis in hybrid rapeseed production. However, this system may face instability issues due to unpredictable environmental conditions [4].

To address the limitations of GMS systems, DuPont-Pioneer implemented a GMS-based seed production technology (SPT) strategy for commercial hybrid seed production [12]. This strategy involves creating an SPT maintainer line by transforming recessive genic male sterility (RGMS). Plants are engineered with a construct that includes three key functional elements: a gene for male sterility to restore fertility, a gene causing pollen lethality to eliminate transgenic pollen, and a fluorescent seed color marker gene for distinguishing seeds during sorting [11, 13]. Self-pollination of the SPT maintainer line yields an equal distribution of maintainer and male-sterile seeds, which can be distinguished using a fluorescent marker. When the maintainer line is cross-pollinated with the male-sterile line, it results in 100% male-sterile seeds. Nonetheless, the risk of transgene escape caused by the incomplete lethality of transgenic pollen raises both safety and efficiency issues for these systems [11–14]. Similar strategies to the SPT system have been developed for constructing transgenic maintainers in maize [11, 15], rice [16–18], and other species [14, 19, 20]. Although these systems involve transgenic maintainer lines, the seeds produced from the male-sterile lines and the resulting hybrid seeds do not carry any transgenic elements.

Generally, the construction of transgenic maintainers is a complicated process involving long processes of laborious backcrossing to introduce natural loss-of-function mutation loci of fertility genes into different backgrounds to generate male-sterile lines. Additionally, biotechnological components must be provided to these sterile lines to facilitate their propagation and sorting with maintainer lines, making the entire process time-consuming and inherently complex. At present, gene editing offers a simple and efficient way to generate precise mutations in many crops, including rapeseed [21–24], which represents a new approach to address this issue. For example, it is challenging to obtain recessive nuclear sterility mutants through either natural spontaneous changes in genetic material or induced alterations via chemical mutagens, because most genes in hexaploid wheat usually contain multiple copies with redundant functions. Researchers have utilized a gene-editing strategy to knock out three *TaNP1* homologous genes that control male sterility in wheat, successfully creating wheat *Tanp1* male-sterile lines and therefore providing a valuable resource for hybrid wheat seed production technology [22]. Although gene editing has been demonstrated to be a strikingly effective and convenient way to develop RGMS lines, its dependency on transgenic methods remains a major bottleneck for gene manipulation in several crops at present [25–27].

In SPT systems, an efficient visual marker is crucial for rapid differentiation between transgenic and non-transgenic plants, thereby reducing the workload. Typically, *DsRed*, a fluorescent protein, is utilized as the primary marker for seed sorting in current SPT systems because of its exceptional stability [11–15]. However, this requires highly accurate fluorescent devices and strong fluorescence signals from seeds, potentially impacting seed sorting accuracy and efficiency. Recent studies have suggested that plant pigment biosynthesis genes are promising alternatives to fluorescent markers [28–31]. For example, the upregulation of the anthocyanin gene *BnaA07.PAP2* promotes anthocyanin synthesis in *B. napus*, resulting in visually identifiable purple seedlings, and thus has operational convenience [32]. Similarly, a synthetic *RUBY* gene is a novel attractive reporter that fuses three key genes for betaine biosynthesis*—CYP76AD1*, *BvDODA1,* and *cDOPA5GT* [28]. Several studies have shown that the expression of *RUBY* can result in vivid red coloration and is readily discernible with the naked eye in maize, tomato, cotton, and foxtail millet, making it a convenient alternative to existing reporters [20, 28, 33]. In foxtail millet, researchers have established an efficient hybrid seed production process by combining the male-sterile *Sipis2* gene with *RUBY* and expressing it in tandem with the *SiPKS2* fertility restorer gene in the same vector [20]. As a result, the seeds of the maintainer line and the male-sterile line can be visually distinguished by their color. This system significantly enhances the efficiency of hybrid seed production. Although the application of these visual markers is expected to work well across different crop species, they have not been tested in *Brassica* plants.

In the present research, we aimed to establish a simple RGMS maintainer system that takes advantage of CRISPR/Cas9 technology and the SPT strategy to rapidly create RGMS lines and SPT maintainer lines directly from different elite rapeseed breeding lines. This system is expected to overcome the problems inherent to traditional GMS systems, including the challenge that lies in the propagation of significant quantities of uncontaminated male-sterile plants and the laborious process of backcrossing to introduce inherent recessive male sterility into various parental genetic backgrounds. Consequently, this approach has significant practical potential for the advancement of hybrid seed breeding and cultivation in rapeseed and other plant species.

## Results

### Efficient generation of RGMS lines by precise editing of the *BnaMS1* and *BnaMS2* genes in different elite parental lines

S45A is a widely utilized RGMS line known for its advantages, such as complete sterility, stable sterility across different environments, widespread restoration, robust heterosis, and high seed production yield [34]. Previous research has shown that the fertility of S45A is regulated by the loss-of-function mutation of two duplicate *BnaCYP704B1-*homologous genes, *BnaMS1* (*BnaA07G0309800ZS*) and *BnaMS2* (*BnaC06G0359600ZS*) [34, 35]*.* Therefore, *BnaMS1* and *BnaMS2* were selected as candidates for the generation of sterile rapeseed lines via gene editing.

**Figure 1:**
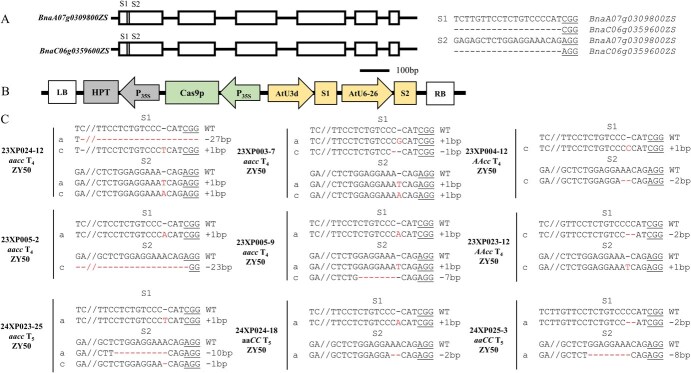
Null mutants of *BnaMS1* and *BnaMS2* by CRISPR/Cas9 technology. (A) *BnaMS1 (BnaA07g0309800ZS)* and *BnaMS2 (BnaC06g0359600ZS)* gene models. There are six exons, denoted by boxes, which are interspersed by five introns, indicated by solid lines. The vertical line within the gene model denotes the specific target site. The target sequences are presented with PAM, the protospacer adjacent motif. (B) The CRISPR/Cas9 system incorporates a hygromycin resistance component, which features the hygromycin phosphotransferase gene under the control of the cauliflower mosaic virus 35S promoter. Additionally, it includes a Cas9 expression module with Cas9 coding sequences regulated by the 35S promoter. Two single-guide RNAs (sgRNAs), labelled S1 and S2, are driven by the AtU3d and AtU6–26 promoters, respectively, which are derived from *Arabidopsis*. (C) The sequences at the sgRNA target sites of homozygous mutants of *BnaMS1* and *BnaMS2* in the T_4_ or T_5_ generation are presented. PAM sequence is underlined, while nucleotide insertions or deletions (indels) are highlighted, with specific details provided on the right side of the sequences. The symbols ‘a’ and ‘c’ denote the mutated alleles of the target gene on *BnaMS1* and *BnaMS2*, respectively. The combinations ‘aaCC,’ ‘AAcc,’ and ‘aacc’ represent homozygous mutations of the target gene in *BnaMS1*, *BnaMS2*, and both loci, respectively.

To assess the efficacy of creating male-sterile lines across various genetic backgrounds via the CRISPR/Cas9 technology, six elite rapeseed breeding lines (ZS11, HS5, ZY50, ZY51, 20P19, and ZS9), which are commonly used as parental lines in current breeding, were selected as the transformation receptors. To induce knockout mutations via CRISPR/Cas9 technology, the *BnaMS1* and *BnaMS2* genes, two specific guide RNAs (sgRNAs), labelled S1 and S2, respectively, were purposefully constructed to specifically target the initial exons of the genes to ensure successful gene disruption and frameshift mutations (Fig. 1A). A binary construct denoted PSH94 was developed, containing two single-guide RNAs (sgRNAs) alongside Cas9 directed by the 2 × 35S promoter, in accordance with established methodologies (Fig. 1B) [36].

The resulting structure was introduced into these receptors separately using the *Agrobacterium*-mediated hypocotyl technique. A total of 97, 102, 88, 4, 3, and 1 independent T_0_-positive transgenic plants were obtained for ZY50, ZY51, 20P19, ZS11, HS5, and ZS9, respectively. The targeted mutations of *BnaMS1* and *BnaMS2* high-throughput tracking of mutations (Hi-TOM) sequencing was used to analyze the target sites (Table S1). There were 68, 65, and 72 target mutants in ZY50, ZY51, and 20P19, respectively, whereas no target mutants were identified in ZS11, HS5, and ZS9. These findings indicated that the widely used *Agrobacterium*-mediated hypocotyl method in rapeseed is only effective for ZY50, ZY51, and 20P19 but is not suitable for the other three elite lines. Therefore, we developed an *Agrobacterium*-mediated epicotyl method for the transformation of ZS11, HS5, and ZS9. Indeed, the new method significantly improved the transformation efficiency, generating 32, 29, and 26 independent T_0_-positive transgenic plants for ZS11, HS5, and ZS9, respectively. Hi-TOM sequencing analysis revealed that the percentages of T_0_-positive transgenic plants edited by ZS11, HS5, and ZS9 were 37%, 37.9%, and 38.4%, respectively. The evaluation of the polymerase chain reaction (PCR) products derived from the specific region of interest in the mutants indicated the presence of mutations in both alleles, showing a combination of heterozygous and homozygous mutations within the targeted genomic region (Table S1).

In order to establish genetically stable plant lines harboring specific mutations, successive generations of self-pollination were conducted following the initial editing of T_0_ plants. The accuracy and presence of the desired mutations in the offspring of T_0_ plants were confirmed through Hi-TOM sequencing analysis (Table S2). A variety of *BnaMS1* and *BnaMS2* single and double homozygous mutants lacking T-DNA insertion were successfully generated from each of these elite parental lines (Fig. 1C, Table S3). As expected, most of the mutations were short (<10 bp). Two larger deletions (23 and 27 bp) were identified in the ZY50 background (23XP024–12 and 23XP005–2). Subsequently, allele-specific INDEL markers were developed on the basis of these larger deletions in the target sequence region, which could be utilized to track these mutations for molecular marker-assisted selection in their progeny (Table S4, Fig. S1). Mutations that are homozygous and located at the designated target regions within *BnaMS1* and *BnaMS2* are expected to cause frameshifts, resulting in the creation of non-functional proteins. Consequently, we selected 23XP024–12 and 23XP005–2 as the RGMS lines for further analysis (Fig. S2).

**Figure 2:**
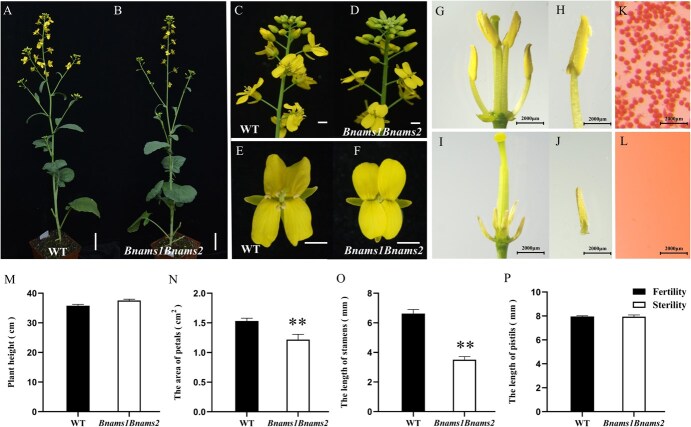
Mutations in the *BnaMS1BnaMS2* gene affect plant fertility. (A–F) Phenotypic comparison of flowers and main branches at the flowering stage between the WT and *Bnams1Bnams2* double mutant plants; scale bar for A–B, 5 cm; scale bar for C–F, 1 cm. (G, H, K) Morphological analysis of petal-free flowers, anthers, and pollen from WT plants. Bars, 2000 μm. (I, J, L) Phenotypes of petal-removed flowers, anthers, and pollen in the *Bnams1Bnams2* double mutant. Bars, 2000 μm. (M–P) Plant height (M), petal area (N), stamen length (O), and pistil length (P) of the WT and *Bnams1Bnams2* double mutant plants. The data are displayed as the mean values with standard errors (*n* ≥ 10). Statistical analysis comparing the WT and double mutant strains was conducted using Student’s *t*-test (**, *P* > 0.01).

These homozygous mutant lines were cultivated in the field alongside their respective wild-type (WT) controls for phenotypic characterization. There were no notable disparities in growth or developmental patterns between the homozygous mutant specimens and their WT counterparts until reaching the flowering stage (Fig. S3A–B, D–E). During flowering, the plant height of the mutants resembled that of the WT plants (Fig. 2A–B, M); however, the petal area of the double mutants was reduced by an average of 8.9% compared with that of the WT plants (Fig. 2C–F, N). Additionally, the stamens were ~4 mm shorter than those of the WT control (Fig. 2G–J, O–P). As expected, all double mutants across different genetic backgrounds displayed complete male sterility with small anthers, shorter filaments, and no pollen in mature anthers (Fig. 2G–L, Table S3). Furthermore, the mutant phenotype remained stable across various environments. Following artificial pollination, the seed-setting rate of the *Bnams1Bnams2* mutants did not significantly differ from that of the WT, indicating normal female fertility (Fig. S3C, F–H). Similarly, the single mutants of *Bnams1* and *Bnams2* exhibited a phenotype comparable to that of the WT, which aligns with prior studies showing that the *BnaMS1* and *BnaMS2* genes have redundant functions in the control of male fertility (Table S3).

In summary, we successfully generated male-sterile lines directly from different elite rapeseed parental lines via gene editing technology. These sterile lines demonstrated stable and complete sterility while maintaining a phenotypically normal appearance, underscoring their excellent breeding potential for producing hybrid seeds.

### Development and characterization of different visual reporters for rapeseed molecular breeding

**Figure 3:**
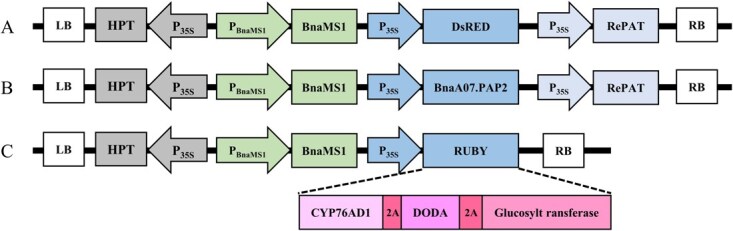
Schematic diagrams of the visual reporter systems used in this study. (A) MSBD cassette-containing construct based on *DsRed*. (B) MSPR cassette-containing construct based on *BnaA07.PAP2*. (C) PMR cassette-featuring construct comprising genes for betalain biosynthesis. LB, left border; RB, right border; HPT, HygR gene; P_35S_, cauliflower mosaic virus (CaMV) 35S promoter; Cas9, SpCas9; P_BnaMS1_, *BnaMS1* native promoter; *BnaMS1*, *BnaMS1* genomic sequence; *DsRed*, gene-encoding red fluorescent protein; *BnaA07. PAP2*, a gene involved in anthocyanin biosynthesis; *RePAT*, a glufosinate ammonium resistance gene. Three genes, namely CYP76AD1, DODA, and glucosyl transferase, play a key role in the biosynthesis of betalains. These genes are associated with the P2A, 2A peptide.

To establish a visual reporter system for rapid and efficient screening of the recessive nuclear male-sterile line S45A at the early growth stage, three SPT cassettes carrying a functional *BnaMS1* gene linked to different reporters (*DsRed*, *BnaA07.PAP2,* and *RUBY*) were constructed and tested in the parent line ZY50. The three reporter gene constructs were designated MSBD, MSPR, and PMR, respectively. These visual reporters, driven by the CaMV35S promoter, were utilized to label transgenic seeds or seedlings. *BnaMS1* under its native promoter restored the male fertility of *Bnams1Bnams2* double mutants (Fig. 3A–C). These constructs were subsequently transformed into ZY50.

For the MSBD construct, a total of 68 positive plants were obtained. Fluorescence microscopy revealed stronger red fluorescence signals in the leaves, buds, flowers, and developing siliques, but the mature seeds of the transgenic plants did not exhibit fluorescence (Fig. S4). Since DsRed-labelled genes are primarily employed to screen mature seeds using fluorescence measuring equipment, *DsRed* is deemed unsuitable for seed sorting in rapeseed.

With respect to the MSPR construct, 72 positive transgenic plants were obtained. In the greenhouse, no phenotypic difference was noticed comparing the transgenic plants and the WT plants (Fig. S5A–D). Considering that anthocyanin synthesis may require ultraviolet (UV) light induction, we planted these plants in the field and observed that the leaves and developing seeds of the MSPR transgenic plants exhibited a distinct purple color (Fig. S5E–K). Quantitative real-time polymerase chain reaction (qRT-PCR) analysis of MSPR transgenic plants showed significantly differential expression of *BnaA07.PAP2* gene under different conditions: it is highly expressed in the field-grown condition, but barely expressed under greenhouse conditions (Fig. S5I). These results further confirmed the regulation of anthocyanin biosynthesis pathways by *BnaA07.PAP2* in rapeseed requires the induction of stronger UV light. Therefore, the *BnaA07.PAP2* is environmentally sensitive, and is not an ideal morphological reporter for plant breeding.

**Figure 4:**
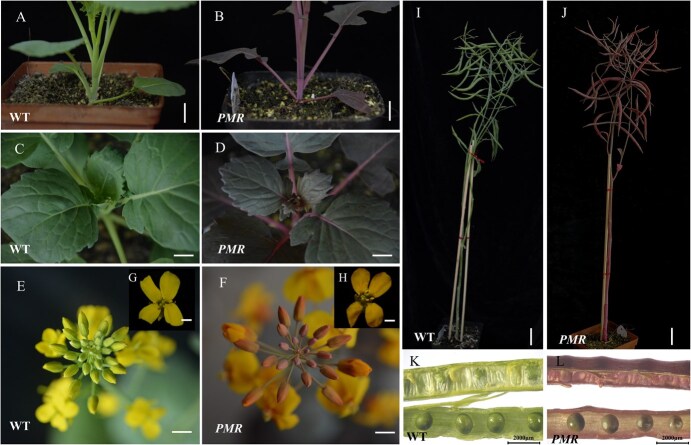
Phenotypic analysis of WT and PMR transgenic rapeseed plants. (A–B) The shoot, (C–D) leaf, (E–F) flower bud, (G–H) flower, (I–J) whole plant at maturity, and (K–L) developing silique morphology of WT (left) and PMR transgenic plants (right). Scale bar for A–J, 1 cm; scale bar for K–L, 2000 μm.

For the PMR construct, we observed deep red pigmentation in calli and shoots during transformation (Fig. S6A–C). A total of 80 positive PMR transgenic plants were subsequently obtained. The transgenic plants exhibited a very distinct and stable red color throughout the entire plant under both greenhouse and field conditions (Fig. 4, Fig. S6D–F). Considering the strong and persistent betaine pigments of the *RUBY* marker, it can serve as a promising marker for tracking gene expression or visualizing transgenic events in rapeseed.

On the basis of the aforementioned evaluation of different reporters, the PMR marker system clearly offers advantages in terms of phenotypic stability and ease of identification, making it an ideal reporter for rapid and efficient screening of recessive nuclear sterile lines at early stages. Consequently, we opted for the PMR-based SPT system for further investigations.

### Screening and characterization of the ideal *PMR* reporter lines

In accordance with the design concepts of the SPT system, the ideal transgenic maintainer should exhibit a conspicuous red color and harbor a single-copy hemizygous T-DNA insertion, containing the PMR reporter and a functional *BnaMS1* gene, alongside homozygous *Bnams1Bnams2* alleles that are Cas9-free. To achieve such maintainers, we initially screened the optimal PMR-based SPT lines characterized by evident red pigmentation and harboring a single hemizygous T-DNA copy from PMR T_0_ transgenic plants generated in the ZY50 background. Following phenotypic evaluation and determination of the segregation ratio of seedling color in their progeny, an ideal T_0_ transgenic event of *PMR-28*.

**Figure 5:**
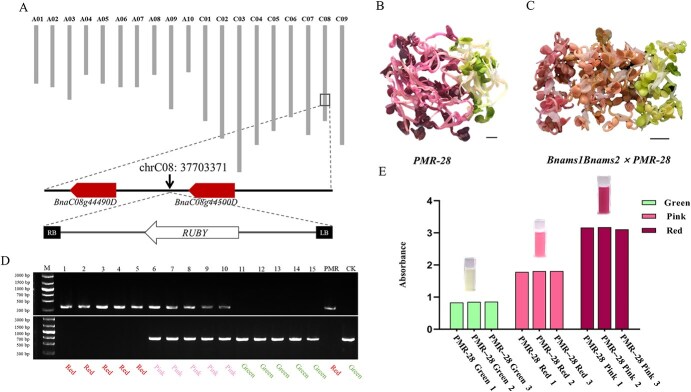
Dosage effect of the homologous/heterozygous insertion of the RUBY gene on seedling color. (A) Localization of T-DNA insertions in the transgenic line *PMR-28*. (B) Segregation of seedling color in the *PMR-28* lines at 2 days after germination. Bar, 1 cm. (C) Segregation of seedling color in *Bnams1Bnams2 × PMR-28* plants at 2 days after germination*.* Bar, 2 cm. (D) Genotypic and phenotypic analysis of *Bnams1Bnams2* × *PMR-28-1* seedlings at 2 days after germination. The codominant PCR marker was used to distinguish the WT, heterozygous, and homozygous genotypes of the T-DNA containing the SPT cassette. (E) The absorbance of betaines on *Bnams1Bnams2* × *PMR-28* seedlings at 2 days after germination*.*

*PMR-28* was chosen as a representative and utilized for further research (Table S5). The betaine pigment of the *PMR-28* seedlings was one of the most pronounced in the T_0_ transgenic lines (Fig. S6F). In the T_1_ generation of *PMR-28*, 32 out of 44 plants were red, and 12 were green, indicating a 3:1 monogenic segregation ratio (χ2_0.05, 1_ = 0.03 < 3.84) (Fig. 5B, Table S5). Subsequently, the T-DNA insertion flanking sequences in *PMR-28* were isolated by reverse PCR and whole-genome resequencing techniques. The T-DNA insertion was positioned at chrC08: 37703371, precisely in the intergenic region between *BnaC08g44490D* and *BnaC08g44500D* (Fig. 5A). A co-dominant PCR marker was created to differentiate the WT, heterozygous, and homozygous genotypes of the T-DNA, relying on the flanking sequence of the T-DNA insertion in *PMR-28* (Table S4). Among the 70 individuals from the F_2_ population resulting from *Bnams1Bnams2 × PMR-28*, the three distinct genotypic types designated by the WT (18 plants), heterozygous (37 plants), and homologous T-DNA insertion (15 plants) exhibited a monogenic segregation ratio of 1:2:1 (χ^2^_0.05, 2_ = 0.03 < 5.99), confirming that *PMR-28* contained a single copy of the SPT cassette (Table S6). Subsequent phenotypic analysis revealed that the red-colored seedlings were heterozygous/homologous genotypes, while the green-colored seedlings were WT genotypes (Fig. 5C–D), confirming that the isolated transgenic T-DNA insertion was responsible for the betaine pigment in the *PMR-28* seedlings.

Interestingly, the red color of these transgenic plants changed to varying degrees, and the plants were grouped into two groups: 37 plants were obviously lighter red (hereafter referred to as pink), and 15 plants were darker red. Genotypic analysis with the T-DNA-specific marker revealed that plants with a pink color had a heterozygous genotype, while plants with a darker red color had a homozygous genotype (Fig. 5C–D). We further quantified the absorbance of extracts from the F_2_ individuals to quantify the betaine content. There were two groups: red seedlings (homozygous individuals) whose absorbance increased and pink seedlings (heterozygous individuals) whose absorbance decreased, indicating a significant dosage effect of the homologous/heterozygous insertion of the *RUBY* gene (Fig. 5E). On the basis of these observations, we selected *PMR-28* as the optimal PMR reporter line for further analysis.

### Development of PMR-based maintainer lines for the *Bnams1Bnams2* male-sterile lines

To develop PMR-based maintainer lines, we crossed the selected *PMR-28* reporter line with *Bnams1Bnams2* double mutants (23XP024–12 and 23XP005–2) created by gene editing in the same genetic background (ZY50). In the F_2_ generation, 107 and 142 red seedlings were selected from each hybridization combination, respectively. Among these, 10 and 18 homozygous *Bnams1Bnams2* double mutants were selected. Subsequently, the heterozygous genotypes of the PMR T-DNA in these double mutants were screened by the segregation ratio of seedling color in their progeny (F_3_ generation). It was observed that the progeny of three and eight lines exhibited obvious red, pink, and green color segregation at a ratio of approximately 1:2:1 for the *23XP024–12 × PMR-28* and *23XP005–2 × PMR-28* combinations, respectively (Table S6). Genotyping with the PMR T-DNA-specific marker further confirmed that these selected lines showing color segregation are heterozygous genotypes. Fertility observations of these individual plants revealed good fertility, indicating that PMR T-DNA can completely restore fertility to that of the *Bnams1Bnams2* double mutants (Table S7). Thus, three and eight lines with the desired genotypes (*ms1/ms2*; *PMR/−*) were selected as the potential PMR-based maintainer lines for each combination, respectively.

The potential PMR-based maintainer lines generated by *23XP024–12* × *PMR-28* (Com024–23) and *23XP005–2* × *PMR-28* (Com005–101) were chosen and evaluated to ascertain their potential. Cross-pollination of the gene-edited *Bnams1Bnams2* male-sterile line (*23XP024–12*) with its potential PMR-based maintainer lines produced 270 seedlings, 141 of which were pink and 129 of which were green, at a 1:1 ratio, as predicted (*χ*^2^_0.05, 1_ = 0.448 < 3.84). Similarly, cross-pollination of the gene-edited *Bnams1Bnams2* male-sterile line (*23XP005–2*) with its potential PMR-based maintainer lines produced 314 seedlings, 149 of which were pink and 165 of which were green, at a 1:1 ratio, as predicted (χ^2^_0.05, 1_ = 0.815 < 3.84) (Fig. S7, Table S8).

Fertility assessment revealed that 100% of the pink seedlings were male-fertile, whereas 100% of the green seedlings were male-sterile, confirming the effectiveness of the PMR-based maintainer lines (Table S9). Subsequent analysis confirmed that the genotypes of the pink and green plants were ‘*ms1/ms2; PMR/−*‘ and ‘*ms1/ms2; −/−*’, respectively, indicating that the heterozygous SPT cassette cosegregated with male fertility and a pink color in the *Bnams1Bnams2* background (Table S9). The separation of seedlings based on their characteristics could be readily and precisely identified within a span of 2–3 days following the onset of germination; thus, this method would be very useful for the rapid differentiation of male-sterile plants from transgenic maintainer plants at the early seedling stage.

Self-pollination of the resulting PMR-based maintainer lines (*ms1/ms2; PMR-28/−*) generated by *23XP024–12* × *PMR-28* showed that the red, pink, and green phenotypes segregated 16:30:19, a ratio of approximately 1:2:1 for monogenic segregation (χ^2^_0.05, 2_ = 0.323 < 5.99). Similarly, self-pollination of the resulting PMR-based maintainer lines (*ms1/ms2; PMR-28/−*) generated by *23XP005–2* × *PMR-28* showed that red, pink, and green phenotypes segregated 20:48:24, a ratio of ~1:2:1 for monogenic segregation (χ^2^_0.05, 2_ = 0.056 < 5.99) (Table S8). Again, red and pink individuals were 100% male-fertile, and all the green individuals also exhibited complete male sterility. Subsequent analysis confirmed that the genotypes of the red, pink, and green plants were ‘*ms1/ms2; PMR/PMR*’, ‘*ms1/ms2; PMR/−*’, and ‘*ms1/ms2; −/−*’, respectively (Table S9).

In conclusion, we successfully developed an ideal PMR-based maintainer line harboring one hemizygous SPT cassette under the *Bnams1Bnams2* genetic background, and the homozygous/heterozygous status of the T-DNA insertion can be effectively differentiated based on its dosage effect. This could potentially improve the effectiveness of propagating recessive genic male-sterile plant lines and producing commercial hybrid rapeseed seeds.

## Discussion

### CRISPR/Cas9 system provides a potent approach to effectively create RGMS lines in elite parental lines of *B. napus*

Generally, the generation of an RGMS line under a new parental breeding background by traditional methods requires extensive laborious backcrossing to introduce natural loss-of-function mutation loci in fertility genes. To overcome this limitation of traditional RGMS systems, we aimed to create recessive male-sterile lines directly from elite rapeseed breeding lines via mutations resulting from harnessing CRISPR/Cas9 in the target genes of the S45A system. This technology relies heavily on the existing genetic transformation technology used in many crops, including rapeseed, which always exhibits a serious genotype-dependent nature and is the major bottleneck for gene editing.

Significant progress has been made in the genetic transformation of *B. napus*. In particular, the *Agrobacterium*-mediated hypocotyl method has been well established and widely used for the genetic transformation of *B. napus* for the past 20 years [37, 38]. Although this transformation protocol has been optimized and has demonstrated acceptable transformation efficiency within a restricted set of genetic variations, the efficacy of the hypocotyl method in elite rapeseed germplasms has proven to be a challenging endeavor [38, 39]. We tested the feasibility of using six different rapeseed elite breeding lines by using the transformation method, which was effective only for the parental lines ZY50, ZY51, and 20P19. Thus, a new genetic transformation method is needed to address these genotype-dependent limitations. Recently, Chu et al [25] devised a method for *Agrobacterium-*mediated transformation utilizing the epicotyl and higher stem (intermodal) segments, which demonstrate genotype-independent characteristics and exhibited differing levels of transformation efficiency. On the basis of this idea, we developed a modified *Agrobacterium*-mediated epicotyl transformation method using 3- to 4-mm-long epicotyls from rapeseed plants grown in light for 15 days as explants; however, most of the transformation procedures and culture media used were the same as those used for the existing hypocotyl transformation methods modified by our laboratory [39]. The addition of 0.5 g/l PVP-40 to M_2_ or M_3_ media was necessary to inhibit the browning of the epicotyl explants. Additionally, the duration of successive cultures during the M_3_ sprouting stage was reduced to 10 days until regenerated plants were obtained from the M_4_ roots. By utilizing the new epicotyl transformation method, the transformation efficiency was significantly improved in different elite rapeseed parental lines, facilitating the successful generation of RGMS lines and thereby shortening the breeding cycle (Table S1). Moreover, epicotyl explants exhibited a more rapid response to tissue culture, manifesting visible shoots within a span of 4–5 weeks, as opposed to the 6–7 weeks required for hypocotyl explants. Although the epicotyl transformation method required eight more days to obtain explants, the total transformation process was 10 days shorter than that of existing hypocotyl transformation methods. Thus, the new epicotyl transformation method developed in the present study is efficient, rapid, and applicable to a diverse array of high-quality rapeseed germplasms.

### RUBY is a novel attractive reporter in *B. napus*

To establish an SPT system, a suitable reporter is crucial for ensuring the effectiveness and precision of seed or seedling sorting. However, there is still a lack of efficient visual markers that are practical for rapeseed. Although DsRed-derived SPT systems have been effectively created and utilized in the context of rice and maize [11–13, 15–17], this reporter seems inefficient for seed sorting in rapeseed. When *DsRed* was driven by the CaMV35S promoter, the red fluorescence signal was nearly undetectable in the fully developed seeds of the genetically modified plants in the ZY50 genetic background (Fig. S4). Similarly, a recent study reported that *DsRed* was not an ideal reporter in foxtail millet because of the difficulty in screening seeds exhibiting red fluorescence while retaining their husks, thereby eliminating the need for a fluorescence detection equipment [20].

Plants synthesize a variety of vibrant pigments, including anthocyanins, betaines, and carotenoids, which have the potential to function as indicators. Anthocyanins, for instance, exhibit vivid red to blue hues, and increasing the synthesis of anthocyanin in rapeseed could result in purple seedlings [32]. Then, we tested the effects of *BnaA07.PAP2*, an endogenous anthocyanin synthesis gene in rapeseed. The MSPR transgenic plants exhibited a distinct purple color that was easily visible to the naked eye (Fig. S5G–K), providing a convenient alternative to existing reporters. However, since the purple color requires the induction of more intense, environmentally sensitive UV light, it can only be used under field-grown conditions.

Betaines are tyrosine-derived pigments, a category of organic compounds derived from plants with a very bright red color [28]. Betaine is an attractive reporter because it is a natural product with health benefits, and the pigment is readily observable to the unaided eye, obviating the necessity for specialized apparatus or chemical interventions [26, 28]. The process of betaine biosynthesis has been extensively researched and involves a series of three enzymatic reactions that transform tyrosine into the brightly colored compound betaine [28, 29]. Given that tyrosine is present in all cells, utilizing anthocyanin biosynthesis pathways as a universal visible indicator would be a practical approach across different crop species. On the basis of this idea, He et al [28] synthesized a synthetic open reading frame referred to as RUBY has the capability, upon expression, to generate the complete set of enzymes necessary for the biosynthesis of betaine. *RUBY* has been shown to be a very effective marker for tracking gene expression or visualizing transgenic occurrences in both Arabidopsis and rice [26, 28]. Subsequently, it was successfully applied to maize, tomato, cotton, and foxtail millet [29, 30, 33], which illustrated that the RUBY reporter exhibits background independence and holds potential for application in various crop species. In the present study, we reported its successful application in rapeseed for the first time. We observed deep red pigmentation as early as callus regeneration during *in vitro* culture (Fig. S6), making it much more convenient to differentiate between transformed callus and untransformed callus. and significantly reducing the workload during transformation. *RUBY* can also be effectively utilized for the identification of individual T-DNA insertion events by examining the segregation ratio of red seedlings to green seedlings in their offspring, which should be 3:1 for single insertions. Using this method, an ideal PMR reporter line, *PMR-28*, which contains a single copy of the SPT cassette, was successfully identified in this study. In addition, we detected a significant dosage effect of the homologous/heterozygous insertion of the single-copy *RUBY* reporter (Fig. 5). On the basis of pigmentation, seedlings of the T_1_ population produced by *PMR-28* were clearly classified into three categories, heterozygous T-DNA insertion (pink), homologous T-DNA insertion (red), and WT (green), exhibiting a monogenic segregation ratio (Fig. 5). This provided powerful tools for visualizing the genotypes of male-sterile plants and PMR-based maintainer lines during the propagation of male-sterile lines and the commercial production of hybrid seeds in rapeseed. Recently, Wang et al [33] reported that *RUBY* driven by the CaMV35S promoter could result in an overabundance of betaine in the leaves, flowers, and fruits of tomato plants leading to a range of harmful characteristics. Although all the tissues throughout the plant life cycle were red, no significant negative effect on betaine accumulation was observed in this study. Hence, it is plausible to posit that RUBY may serve as a more appealing reporter compared to the previously mentioned reporters in rapeseed and other crop varieties.

### Establishment of a new convenient two-line system for hybrid seed production in *B. napus*

**Figure 6:**
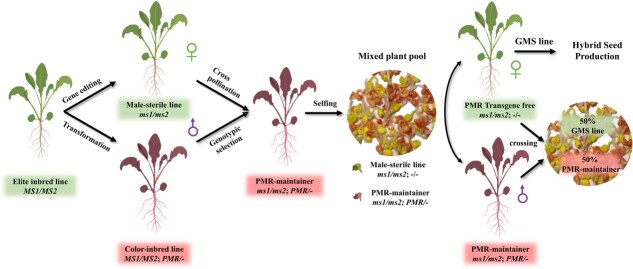
Establishment of a new convenient two-line system for hybrid seed production in *B. napus*. The non-transgenic RGMS lines from *Bnams1Bnams2* double mutants and the ideal PMR reporter lines sharing the same background were pyramided together by crossing. Then, PMR-based maintainer lines harboring *Bnams1Bnams2* double mutants (*ms1/ms2; PMR/−*) were generated in the process of cross-pollination between the male-sterile line and the resulting maintainer line, 50% of the maintainer seedlings propagated, and 50% of the male-sterile seedlings were sorted on the basis of seedling color.

Here, we established a straightforward and efficient PMR-based SPT strategy that takes advantage of CRISPR/Cas9 and SPT technology to directly create RGMS lines and SPT maintainer lines among elite rapeseed breeding lines (Fig. 6). In this approach, two different vectors were transformed separately into the same genetic background: one to generate RGMS by CRISPR/Cas9 and the other to restore fertility and seedling sorting (Fig. 6). The resulting non-transgenic RGMS lines and the ideal PMR reporter lines were pyramided together by crossing. Then, PMR-based maintainer lines harboring *Bnams1Bnams2* double mutants (*ms1/ms2; PMR/−*) were generated. By cross-pollinating the resulting maintainer line with the male-sterile line, 50% of the maintainer seedlings propagated, and 50% of the male-sterile seedlings were sorted depending on seedling color. This method offers a cost-efficient and effective means of distinguishing male-sterile plants within segregating populations in the context of male-sterile line propagation and the generation of hybrid seeds in rapeseed. The current strategy, which is based on the creation of male-sterile lines and SPT cassettes, could be enhanced through a singular genetic modification process involving Agrobacterium carrying two vectors, a technique that has proven successful in maize and foxtail millet [10, 11, 20]. This method is entirely viable within the framework of our current system and will enable the concurrent production of RGMS lines and PMR-based maintainer lines in rapeseed.

The established PMR-based two-line system offers several advantages for the production of hybrid seeds. First, the sterility of RGMS is controlled by recessive nuclear genes, which could be convenient for the direct generation of male-sterile lines from elite breeding lines via our improved *Agrobacterium*-mediated CRISPR/Cas9 mutagenesis method, making the entire process simple and efficient. Second, rapeseed germplasms containing the corresponding dominant fertility genes have the potential to function as a restorer line for RGMS-targeted mutants. Therefore, this research offers hybrid breeding programs with an extensive range of germplasms to utilize as paternal lines. Third, it is easy to differentiate the WT, homozygous, and heterozygous status of the SPT cassette at the seedling stage according to the *RUBY* reporter dosage effect, greatly reducing the labor required for propagation of male-sterile lines and the production of commercial hybrid seeds. Additionally, our system should have fewer environmental and health concerns because the *RUBY* reporter is a health product in plants and because both the male-sterile lines and the hybrid seeds obtained do not possess any transgenic components.

## Materials and methods

### Plant materials and growth conditions

Elite semi-winter *B. napus* varieties, including ZS11, HS5, ZY50, ZY51, 20P19, and ZS9， served as recipients of the genetic transformation process. Both the transgenic and WT plant lines were grown in a greenhouse with a controlled environment, featuring 16 h of light and 8 h of darkness, and a temperature set to 22°C. The *Bnams1Bnams2* mutant plants (Table S3) were cultivated during the winter season of 2023–24 in a specified field at Huazhong Agricultural University in Wuhan, China. Standard breeding practices were used for field management.

### Vector construction

Two sgRNAs were developed to simultaneously target the coding sequences of *BnaMS1* and *BnaMS2* to disrupt their function. The *BnaMS1* gene sequence was amplified from ZS11 and then fused together with *BamH*I/*Pst*I before being inserted into the pMDC163 vector for complementation. Additionally, the target sequence of exogenous *BnaMS1* was modified using a synonymous codon replacement approach resulting in a vector named pMDC163-MS1. Next, the *DsRed* fluorescent protein and the glufosinate ammonium resistance gene *RePAT* were introduced into pMDC163-MS1 to obtain the reporter vector MSBD. Similarly, the MSPR expression element, including the anthocyanin biosynthesis gene *BnaA07.PAP2* and glufosinate ammonium resistance gene *RePAT*, the PMR expression element including betaine biosynthesis gene *RUBY*, were also cloned separately to be inserted into pMDC163-MS1 vector (Fig. 3). The resulting CRISPR/Cas9 and reporter vectors were then introduced into *GV3101* for genetic transformation. The aforementioned primers are detailed in Table S3.

### Hypocotyl and epicotyl transformation method

The hypocotyls were transformed using *Agrobacterium* based on the method outlined by Zhou et al [39] with hypocotyl segments. For epicotyl transformation, 3- to 4-mm-long epicotyls of rapeseed were grown in light for 15 days as explants, whereas most of the transformation procedures and culture media used were the same as those used for the existing hypocotyl transformation methods modified by our laboratory [39]. Specifically, the addition of 0.5 g/l PVP-40 to M_2_ or M_3_ media was required to inhibit the browning of the epicotyl explants. Additionally, the duration of successive cultures during the M_3_ sprouting stage was reduced to 10 days until regenerated plants were obtained from the M_4_ roots.

### DNA extraction, identification, and genotyping of transgenic plants

Initially, DNA from the genome was extracted from fresh leaves of genetically modified plants using the cetyltrimethyl ammonium bromide (CTAB) method. Subsequently, PB-L/PB-R primers were employed to detect the T-DNA (Table S4) in order to validate the successful generation of the regenerated plants through the CRISPR system. Subsequently, an investigation was conducted to ascertain whether the sequences of the specific genes were altered, by confirming genomic fragments encompassing the targeted regions of *BnaMS1* and *BnaMS2* employing the Hi-TOM platform [40]. The sequencing data obtained were then analyzed using an online tool to monitor mutations at the designated sites (http://www.hi-tom.net/hi-tom/).

### Isolation of the flanking sequences of T-DNA insertions in *B. napus* transformants

Inverse PCR [41] and whole-genome resequencing techniques [42] were used to isolate the flanking sequences of the location where T-DNA is inserted into the genome in transgenic lines containing a single copy of the SPT cassette.

### Phenotypic characterization

The *DsRed* fluorescence emitted by seeds and other tissues was visually investigated and recorded using a Leica M165FC fluorescence microscope (Leica, Weztlar, Germany). The excitation and emission center wavelengths for *DsRed* were determined to be 558 and 583 nm, respectively. To assess pollen viability, the pollen grains were extracted from crushed anthers using forceps and subsequently stained with a 1% acetocarmine solution (comprising 45% acetate and 1 g carmine). Fertile pollen grains exhibited a red coloration due to normal starch accumulation, while sterile pollen grains appeared colorless and lacked starch accumulation. The acetocarmine-stained pollen was then visualized under the Leica M165FC microscope. The betaine levels were determined by processing 2.5 g of fresh germ tissue with 7 ml of ddH_2_O, quartz sand, and CaCO_3_, followed by thorough grinding, filtration, and centrifugation. The absorbance of the supernatant obtained was recorded at 535 nm. Various yield-related characteristics， such as plant height, length of siliques, seed count per silique, weight of 1000 seeds, and seed setting rate were quantified according to established protocols. [43].

### RNA extraction and expression analysis

Leaf samples were processed to isolate total RNA using TRIzol reagent (Invitrogen). The synthesis of first-strand cDNA was then performed with Superscript III RT (Invitrogen) using 1 μg of RNA. qRT-PCR was carried out with SYBR Green PCR Master Mix (ABI), using BnaActin7 as an internal control. Relative expression levels were analyzed using the 2^^(-ΔCt)^ method, and standard deviation (SD) was computed from three independent biological replicates. Primer sequences for qRT-PCR are listed in Table S4.

## Supplementary Material

Web_Material_uhae270

## Data Availability

All data supporting the findings of this study are available within the paper and the supplementary data.
