# Supplementary material for: Targeted mutation of BnaMS1/BnaMS2 combined with the RUBY reporter enables an efficient two-line system for hybrid seed production in Brassica napus
Source: Hortic Res. 2024 Sep 25;12(1):uhae270. doi: 10.1093/hr/uhae270 (PMC11783330; doi:10.1093/hr/uhae270)
Supplement: Web_Material_uhae270 [file web_material_uhae270.zip › Supporting figures.docx]

**

**

**
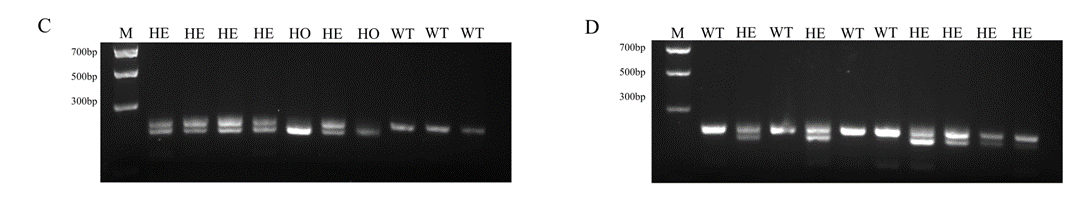
**

**Figure S1. Two larger deletions (23 bp and 27 bp) were identified in ZY50 background (23XP024-12 and 23XP005-2).** (A) Sanger sequencing of the S1 and S2 targets of *BnaMS1.* (B) Sanger sequencing of the S1 and S2 targets of *BnaMS2*. Insertions and deletions are marked with red triangles and target site sequences are marked with purple boxes. (C) Identification of WT, heterozygous and homozygous mutations by the allele-speciﬁc INDEL marker in the progeny of 23XP024-12. (D) Identification of WT, heterozygous and homozygous mutations by the allele-speciﬁc INDEL marker in the progeny of 23XP005-2. HE, heterozygous; HO, homozygous; WT, wild-type.

**A**

**23XP024-12 aacc T_4_**

>A07

BnaMS1 MSMWIVLACM LTSWIFLHRW GQRNKRGPKT WPLVGAAIEQ LTNFDRMHDW LVEYLYDSRT 60

aa MSMWIVLACM LTSWIKRGPK TWPLVGAAIE QLTNFDRMHD WLVEYLYDSR TVVVPMPFTT 60

BnaMS1 VVVPMPFTTY TYIADPINVE HVLKTNFSNY PKGETYHSYM EVLLGDGIFN SDGELWRKQR 120

aa YTYIADPINV EHVLKTNFSN YPKGETYHSY MEVLLGDGIF NSDGELWRNT EENREFRVCF 120

BnaMS1 KTASFEFASK NLRDFSTVVF KEYSLKLFSI LCQASFKDQQ VDMQELLMRM TLDSICKVGF 180

aa QESQRLQYCS V* 131

BnaMS1 GVEIGTLAPD LPENRFAKAF DTANIIVTLR FIDPLWKMKK YLNIGSEALL GKSIKVVDDF 240

aa

BnaMS1 TYSMIRRRKT EILEAQKSPS NNIKMKHDIL SRFIEISDDP DSKSTEKSLR DIVLNFVIAG 300

aa

BnaMS1 RDTTATTLTW AIYMIMMNEH VAEKLCSELQ ELEREKAEET NTPLRQYDTE DFKSFNERVT 360

aa

BnaMS1 QFAGMLSYDS LGKLHYLHAV VTETLRLYPA VPQDPKGVLE DDILPNGTKV KAGGMVTYVP 420

aa

BnaMS1 YSMGRMEYNW GSDAATFKPE RWLKDGMFQN ASPFKFTAFQ AGPRICLGKD SAYLQMKMAM 480

aa

BnaMS1 AILCRFYKFH LVPNHPVKYR MMTILSMAHG LKVTVSRRS* 519

aa

>C06

BnaMS2 MSMWIVLACM VTSWIFLHRW GQRNKRGPKT WPLVGAAIEQ LTNFDRMHDW LVEYLYDSRT 60

cc MSMWIVLACM VTSWIFLHRC GTEEQERSQD MAFGRSSH* 38

BnaMS2 VVVPMPFTTY TYIADPINVE HVLKTNFSNY PKGETYHSYM EVLLGDGIFN SDGELWRKQR 120

cc

BnaMS2 KTASFEFASK NLRDFSTVVF KEYSLKLFSI LCQASFKDQQ VDMQELLMRM TLDSICKVGF 180

cc

BnaMS2 GVEIGTLAPE LPENRFAKAF DTANIIVTLR FIDPLWKIKK YLNIGSEALL GKSIKVVDDF 240

cc

BnaMS2 TYSMIRRRKA EILEAQKSPS NNTKMKHDIL SRFIEISDDP DSKSTEKSLR DIVLNFVIAG 300

cc

BnaMS2 RDTTATTLTW AIYMIMMNEH VAEKLCSELQ ELEREKAEET NTPLRQYDTE DFKSFNERVT 360

cc

BnaMS2 QFAGMLSYDS LGKLHYLHAV VTETLRLYPA VPQDPKGVLE DDILPNGTKV KAGGMVTYVP 420

cc

BnaMS2 YSMGRMEYNW GSDAAMFKPE RWLKDGMFQN ASPFKFTAFQ AGPRICLGKD SAYLQMKMAM 480

cc

BnaMS2 AILCRFYRFH LVPNHPVKYR MMTILSMAHG LKVTVSRRS* 519

cc

**B**

**23XP005-2 aacc T4**

>A07

BnaMS1 MSMWIVLACM LTSWIFLHRW GQRNKRGPKT WPLVGAAIEQ LTNFDRMHDW LVEYLYDSRT 60

aa MSMWIVLACM LTSWIFLHR* 19

BnaMS1 VVVPMPFTTY TYIADPINVE HVLKTNFSNY PKGETYHSYM EVLLGDGIFN SDGELWRKQR 120

aa

BnaMS1 KTASFEFASK NLRDFSTVVF KEYSLKLFSI LCQASFKDQQ VDMQELLMRM TLDSICKVGF 180

aa

BnaMS1 GVEIGTLAPD LPENRFAKAF DTANIIVTLR FIDPLWKMKK YLNIGSEALL GKSIKVVDDF 240

aa

BnaMS1 TYSMIRRRKT EILEAQKSPS NNIKMKHDIL SRFIEISDDP DSKSTEKSLR DIVLNFVIAG 300

aa

BnaMS1 RDTTATTLTW AIYMIMMNEH VAEKLCSELQ ELEREKAEET NTPLRQYDTE DFKSFNERVT 360

aa

BnaMS1 QFAGMLSYDS LGKLHYLHAV VTETLRLYPA VPQDPKGVLE DDILPNGTKV KAGGMVTYVP 420

aa

BnaMS1 YSMGRMEYNW GSDAATFKPE RWLKDGMFQN ASPFKFTAFQ AGPRICLGKD SAYLQMKMAM 480

aa

BnaMS1 AILCRFYKFH LVPNHPVKYR MMTILSMAHG LKVTVSRRS* 519

aa

>C06

BnaMS2 MSMWIVLACM VTSWIFLHRW GQRNKRGPKT WPLVGAAIEQ LTNFDRMHDW LVEYLYDSRT 60

cc MSMWIVLACM VTSWIFLHRW GQRNKRGPKT WPLVGAAIEQ LTNFDRMHDW LVEYLYDSRT 60

BnaMS2 VVVPMPFTTY TYIADPINVE HVLKTNFSNY PKGETYHSYM EVLLGDGIFN SDGELWRKQR 120

cc VVVPMPFTTY TYIADPINVE HVLKTNFSNY PKGETYHSYM EVLLGDGIFN SEENREFRIC 120

BnaMS2 KTASFEFASK NLRDFSTVVF KEYSLKLFSI LCQASFKDQQ VDMQELLMRM TLDSICKVGF 180

cc FQES* 124

BnaMS2 GVEIGTLAPE LPENRFAKAF DTANIIVTLR FIDPLWKIKK YLNIGSEALL GKSIKVVDDF 240

cc

BnaMS2 TYSMIRRRKA EILEAQKSPS NNTKMKHDIL SRFIEISDDP DSKSTEKSLR DIVLNFVIAG 300

cc

BnaMS2 RDTTATTLTW AIYMIMMNEH VAEKLCSELQ ELEREKAEET NTPLRQYDTE DFKSFNERVT 360

cc

BnaMS2 QFAGMLSYDS LGKLHYLHAV VTETLRLYPA VPQDPKGVLE DDILPNGTKV KAGGMVTYVP 400

cc

BnaMS2 YSMGRMEYNW GSDAAMFKPE RWLKDGMFQN ASPFKFTAFQ AGPRICLGKD SAYLQMKMAM 460

cc

BnaMS2 AILCRFYRFH LVPNHPVKYR MMTILSMAHG LKVTVSRRS* 499

cc

**Figure S2. The predicted amino acid sequences of homozygous *BnaMS1BnaMS2* mutants in the T_4_ generation.** 23XP024-12 (A) and 23XP005-2 (B) represent *BnaMS1BnaMS2* double-homozygous mutant lines*.* Stars indicate stop codons, and numbers indicate amino acid positions; red letters indicate frame-shift amino acids; “-” indicates deletion of amino acid; “*aa*” and “*cc*” represent the homozygous mutated alleles of the target gene on *BnaMS1* and *BnaMS2*, respectively; “*aacc*” represents homozygous mutations of the target gene in *BnaMS1*, *BnaMS2* and both copies, respectively.


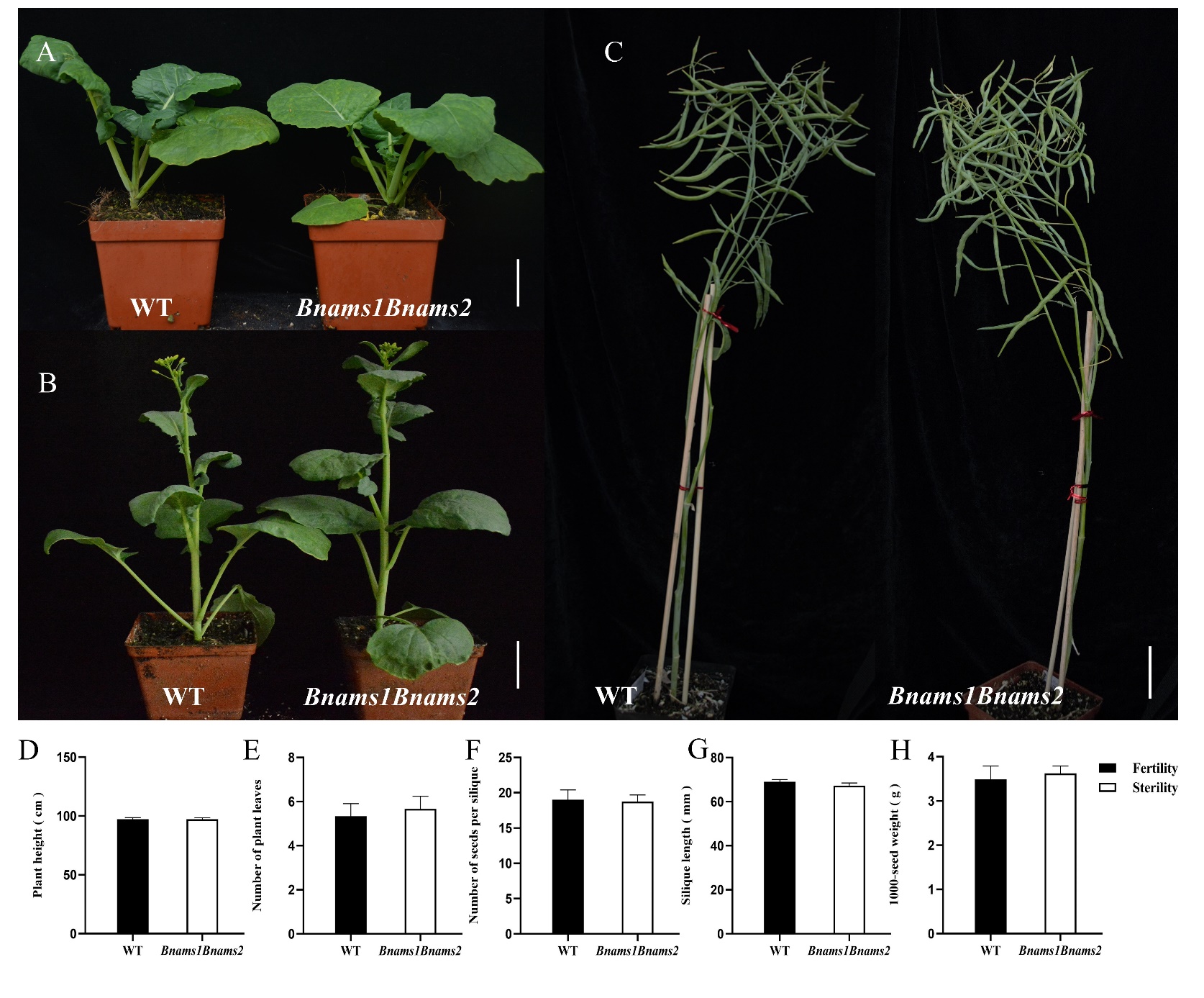


**Figure S3.** **The different growth stages of the wild-type and *Bnams1Bnams2* homozygous mutants.** (A-C) Growth phenotypes of wild-type plants and *Bnams1Bnams2* homozygous mutants at the seedling, vegetative and maturity stage. Bars, 5 cm. (D-H) Number of leaves, plant height, number of seeds per silique, silique length and 1000-seed weight of the wild-type (left) and *Bnams1Bnams2* homozygous mutant (right) plants. The data are presented as the means ± SEs (*n* ≥ 10); Student’s t test was used for the statistical analysis of differences between the sterile plants and fertile plants (***P* > 0.01).


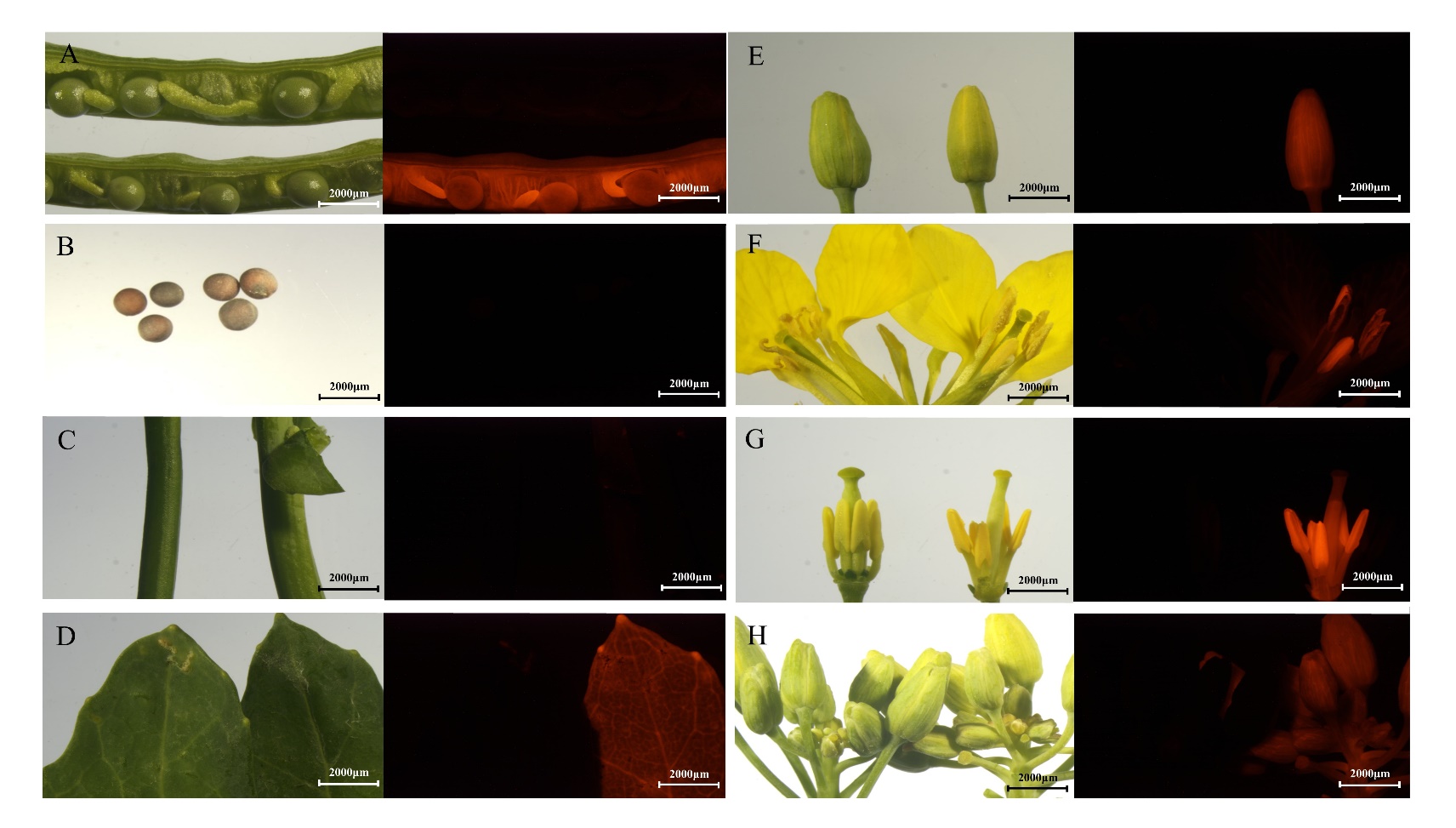


**Figure S4. Expression of the red fluorescent protein DsRED in various tissues of *B. napus.*** (A) Developing silique. (B) Mature seeds. (C) Shoot. (D) Leaf. (E) Individual bud. (F) Flower. (G) Petal-removed flower. (H) Flower buds. Bars, 2000μm.

**
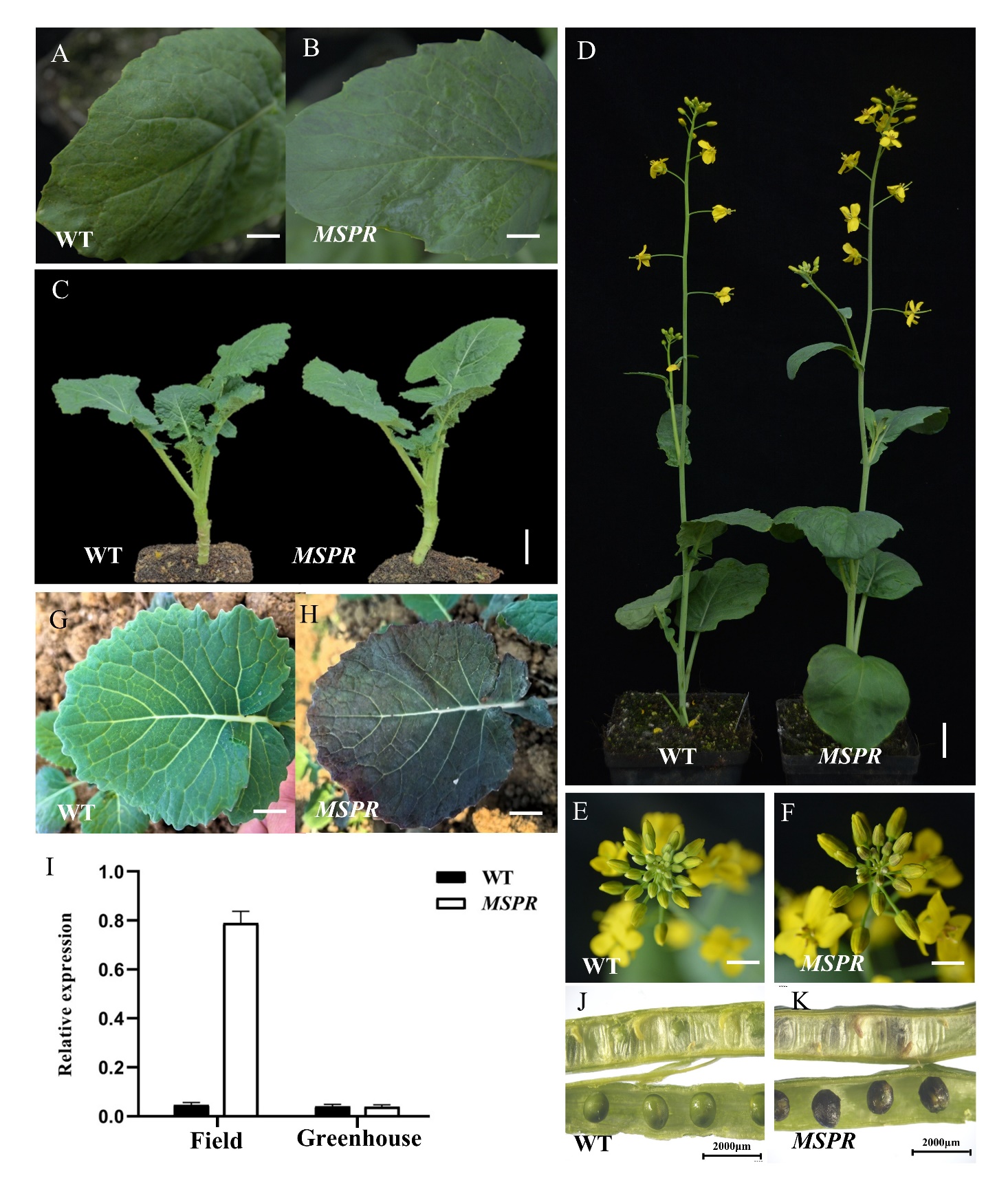
**

**Figure S5. Phenotypic analysis of WT and MSPR transgenic rapeseed.** (A-B) The leaf and (C-D) whole plant morphology of the WT (left) and MSPR transgenic plants (right) in the greenhouse. Bars, 2 cm. (E-F) The flower bud and (G-H) leaf morphology of WT (left) and MSPR transgenic (right) plants in the field. Bars, 1 cm. (I) Gene expression levels of *BnaA07.PAP2* analysis under greenhouse and field conditions by qRT-PCR analysis. (J-K) The developing silique morphology of the WT (left) and MSPR transgenic (right) plants in the field. Bars, 2000μm.


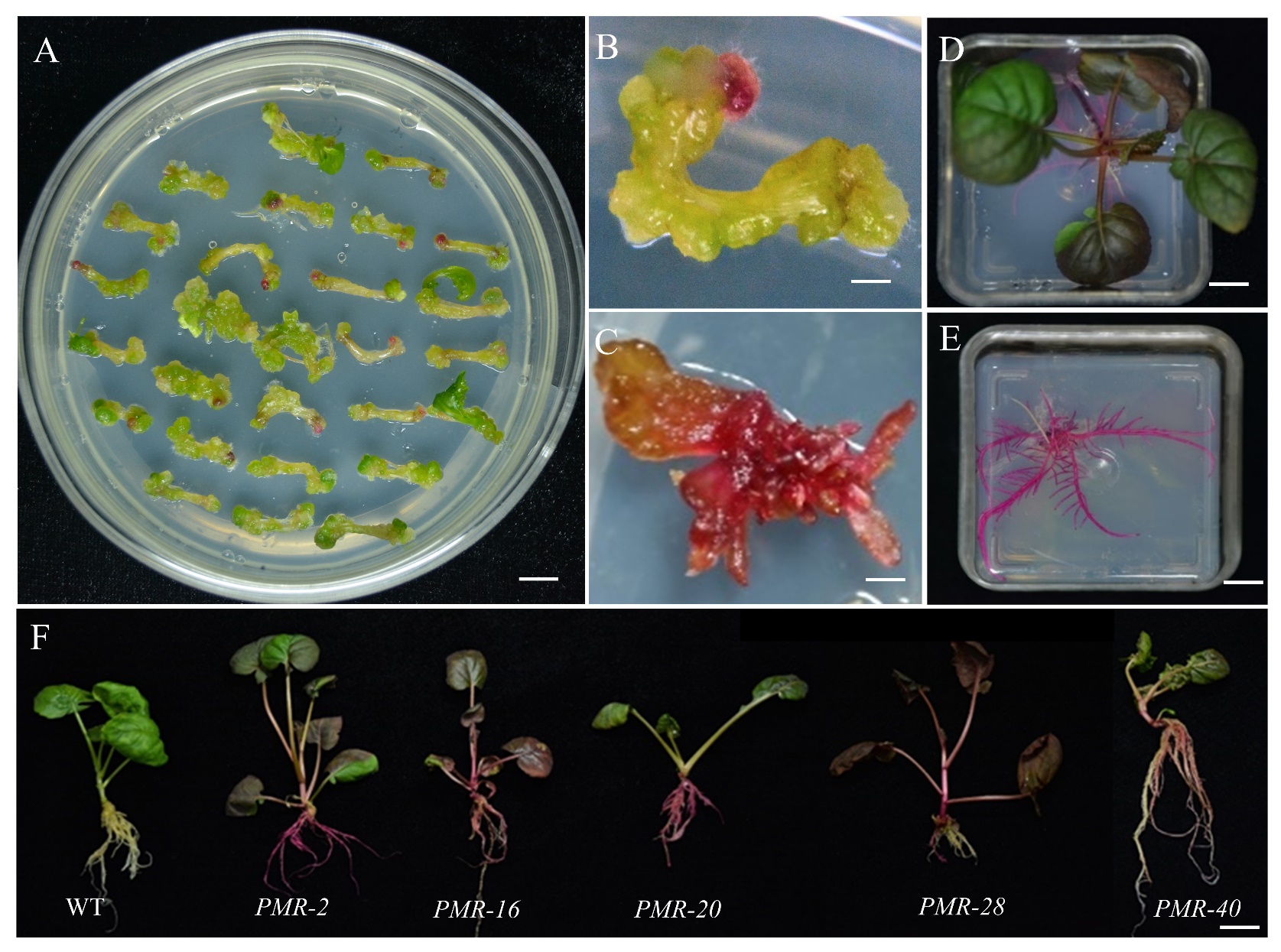


**Figure S6. T_1_ transgenic segregation analysis of PMR lines with a single DNA insertion.**

(A) Transgenic (red) and nontransgenic (green) calli at 30 days after infection; (B-C) transgenic calli, brightfield. (E) Phenotypic T_0_ generation transgenic plants of the PMR lines. Scale bar for A, 1 cm; scale bar for B-C, 2 mm; scale bar for E, 2 cm.

**
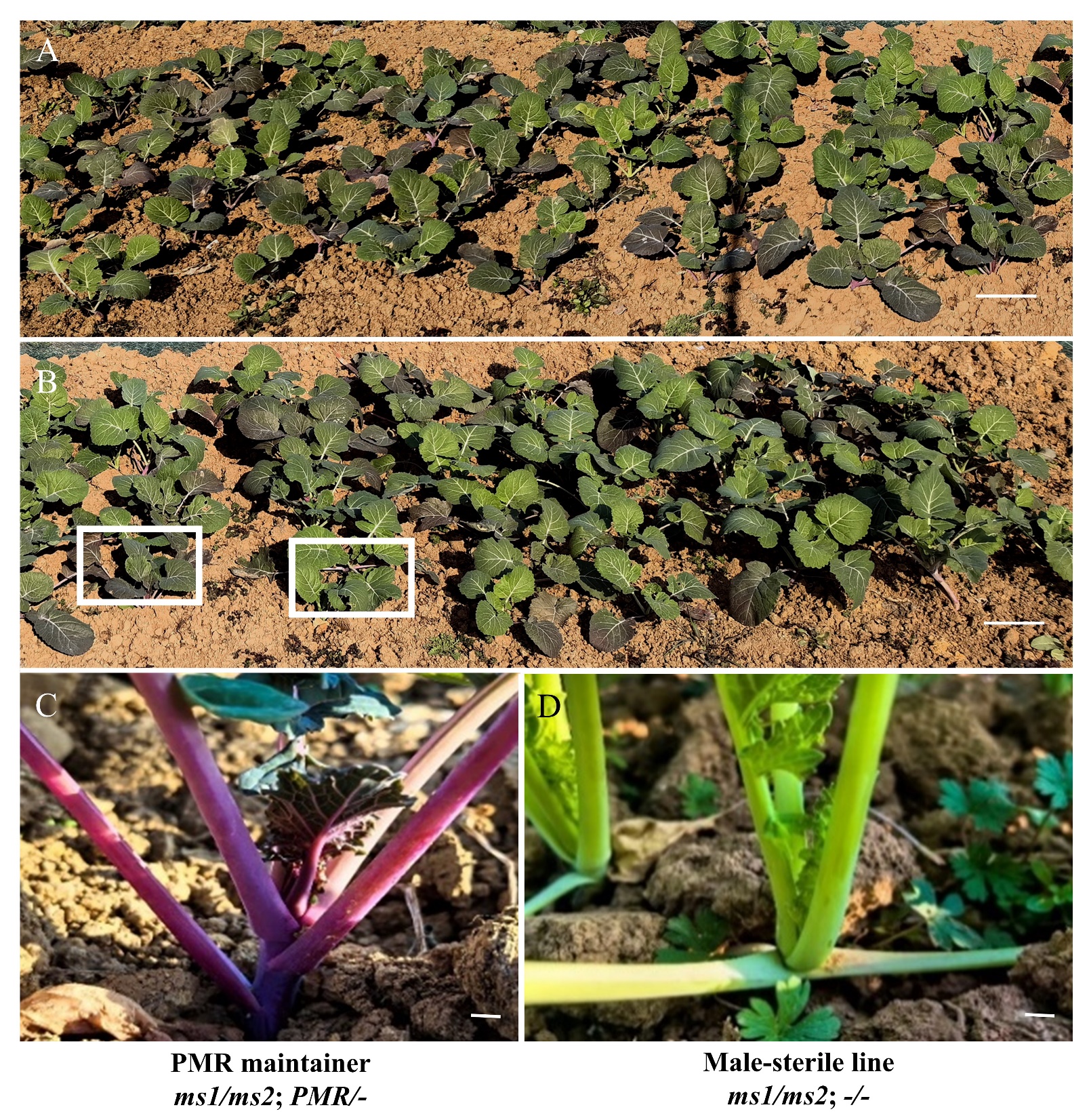
**

**Figure S7. Cross-pollination of the gene-edited *Bnams1Bnams2* male-sterile line (23XP024-12 and 23XP005-2) by its potential PMR-based maintainer lines.** (A-B) Field materials for producing PMR-based maintainer lines and male-sterile lines. Bars, 10 cm. (C) PMR-based maintainer line. (D) Male-sterile line. Bars, 1 cm.
